# Can Nitrogen Excretion of Dairy Cows Be Reduced by Genetic Selection for Low Milk Urea Nitrogen Concentration?

**DOI:** 10.3390/ani11030737

**Published:** 2021-03-08

**Authors:** Hewa Bahithige Pavithra Chathurangi Ariyarathne, Martin Correa-Luna, Hugh Blair, Dorian Garrick, Nicolas Lopez-Villalobos

**Affiliations:** 1 School of Agriculture and Environment, Massey University, Palmerston North 4442, New Zealand; H.Blair@massey.ac.nz (H.B.); D.Garrick@massey.ac.nz (D.G.); N.Lopez-Villalobos@massey.ac.nz (N.L.-V.); 2 INRA, UE Herbipôle, F-63122 Saint-Genès-Champanelle, France; martin.correa.luna@gmail.com

**Keywords:** four-pathways of selection, progeny testing, milk urea nitrogen, urinary nitrogen, dairy cows

## Abstract

**Simple Summary:**

New Zealand dairy cows predominantly feed on pasture with protein to energy ratios well in excess of requirements and consequently their urine has a substantially greater concentration of nitrogen compared to cows fed indoors on total mixed rations. The nitrogen excreted directly onto land decompose into nitrous oxide and ammonium, causing global warming and freshwater pollution, respectively. One of the strategies to reduce the environmental contamination is genetic selection for reduced nitrogen excretion. The nitrogen excreted in urine is an expensive trait to measure in cows grazed outdoors. One possible approach to reduce the concentration of nitrogen excreted in urine is by selection for reduced urea nitrogen concentration in milk, which is readily measurable. The primary objective of this study was to quantify the likely correlated responses for milk production and liveweight traits in a selection index including breeding values for concentration of milk urea nitrogen with different relative emphasis in order to evaluate the usefulness of milk urea nitrogen for the purpose of reducing nitrogen excretion. Although a per cow reduction in urinary nitrogen excretion was predicted through selection in this study, the reduction of excretion is negligible at the whole farm level due to the influence of stocking rate, measured as the number of cows grazed per hectare, on the per hectare nitrogen excretion. Therefore, selection for low concentration of milk urea nitrogen is unlikely to be effective in reducing nitrogen excretion of dairy cows in New Zealand.

**Abstract:**

The objectives of this study were two-fold. Firstly, to estimate the likely correlated responses in milk urea nitrogen (MUN) concentration, lactation yields of milk (MY), fat (FY) and crude protein (CPY) and mature cow liveweight (LWT) under three selection scenarios which varied in relative emphasis for MUN; 0% relative emphasis (MUN_0%_: equivalent to current New Zealand breeding worth index), and sign of the economic value; 20% relative emphasis positive selection (MUN_+20%_), and 20% relative emphasis negative selection (MUN_−20%_). Secondly, to estimate for these three scenarios the likely change in urinary nitrogen (UN) excretion under pasture based grazing conditions. The predicted genetic responses per cow per year for the current index were 16.4 kg MY, 2.0 kg FY, 1.4 kg CPY, −0.4 kg LWT and −0.05 mg/dL MUN. Positive selection on MUN in the index resulted in annual responses of 23.7 kg MY, 2.0 kg FY, 1.4 kg CPY, 0.6 kg LWT and 0.10 mg/dL MUN, while negative selection on MUN in the index resulted in annual responses of 5.4 kg MY, 1.6 kg FY, 1.0 kg CPY, −1.1 kg LWT and −0.17 mg/dL MUN. The MUN_−20%_ reduced both MUN and cow productivity, whereas the MUN_+20%_ increased MUN, milk production and LWT per cow. Per cow dry matter intake (DMI) was increased in all three scenarios as milk production increased compared to base year, therefore stocking rate (SR) was adjusted to control pasture cover. Paradoxically, ten years of selection with SR adjusted to maintain annual feed demand under the MUN_+20%_ actually reduced per ha UN excretion by 3.54 kg, along with increases of 63 kg MY, 26 kg FY and 16 kg CPY compared to the base year. Ten years of selection on the MUN_0%_ index generated a greater reductions of 10.45 kg UN and 30 kg MY, and increases of 32 kg FY and 21 kg CPY per ha, whereas the MUN_−20%_ index reduced 14.06 kg UN and 136 kg MY with increases of 32 kg FY and 18 kg CPY compared to base year. All three scenarios increased partitioning of nitrogen excreted as feces. The selection index that excluded MUN was economically beneficial in the current economic circumstances over selection indices including MUN regardless of whether selection was either for or against MUN. There was no substantial benefit from an environmental point of view from including MUN in the Breeding Worth index, because N leaching is more a function of SR rather than of individual cow UN excretion. This study demonstrates that attention needs to be paid to the whole system consequences of selection for environmental outcomes in pastoral grazing circumstances.

## 1. Introduction

Dairying in New Zealand is pasture based with stocking rate and mating dates chosen to achieve concordance between predicted pasture supply and feed demand of the herd. The feeding system is highly dependent on seasonal pasture supply, and therefore, the stocking rate of cows is managed to achieve the optimal pasture utilization for producing milk and milk solids [1]. The level of supplementary feeding is low [2] compared to the levels found in indoor feeding systems such as those in North America or Europe [3]. Crops may be used for offsetting pasture shortages in winter or any summer months with drought. Fresh pasture contains high quantities of crude protein relative to energy, therefore cows grazing pastures without energy supplementation involuntarily consume excessive quantities of protein, thereby greatly exceeding their requirements [4]. When the consumed pasture is processed in the rumen, ruminally degradable proteins are broken down to amino acids and ammonia. The ammonia is transported to the liver and converted to urea which enters the blood stream and a small proportion is diffused into the milk, some is recycled back to the rumen via saliva, while the bulk of the urea is transported to the kidneys for excretion in urine [5].

Milk urea nitrogen (MUN) concentration has been proposed as a diagnostic tool for protein relative to energy feeding of animals [6,7]. Protein feeding of cows is an expensive component of the diet relative to energy in many countries. Further, the process of converting rumen derived ammonia to urea in the liver is energy [8] and protein [9] demanding. Some researchers have pointed out direct negative effects of MUN on reproductive performance [10,11] as a result of increased blood urea nitrogen concentrations and decreased uterine pH [12]. Guinot-Thomas [13] reported that cheese manufacture can also be compromised given the greater coagulation time required for milk with higher MUN. A major concern from the environmental point of view is that MUN may be positively associated with the amount of urinary nitrogen (UN) excreted by the cow [14]. Nitrogen (N) that enters the environment as UN breaks down to ammonia and nitrous oxide at the site of the urine patch making it a source of water and air pollution. Averaged across the year, 20% of UN load is typically leached through the soil [15].

There are several options that could contribute to reductions in UN. Feeding cows with a diet balanced for ruminally degradable protein (RDP) and energy content is an appealing option if energy supplementation is cost-effective and practical. In a balanced diet, ammonia produced during RDP metabolism in the rumen can be readily captured by rumen microbes and utilized for microbial protein synthesis which generates usable protein for the cow [16]. Microbial protein synthesis is highly sensitive to the amount of fermentable energy in the diet [17]. If the diet contains insufficient fermentable energy then the rumen microbes are inefficient at capturing the ammonia, hence the generated ammonia is converted into urea in the liver and excreted in urine as an un-utilized form of protein. In New Zealand pasture systems, a balanced diet would require energy supplementation which is expensive and intensive in terms of increasing demand for labor, feed storage facilities and feeding equipment. Feeding cows with less RDP [18] and adjusting the protein supply in diet to satisfy the protein requirement of the cow [16] are among other feeding management strategies discussed in literature to reduce N wastage.

Retention of replacement heifers sired by bulls selected for low MUN has been suggested as a useful option for reducing N excretion by Beatson et al. [19]. They assumed that New Zealand cows competing with each other for voluntary intake of pasture had the same relationship between MUN and UN as overseas cows fed with total mixed rations (TMR) and assumed that reducing MUN through breeding has a similar effect on UN to reducing MUN through feeding. This presumably reflects improved N utilization efficiency of cows, identifying cows that partition more of their dietary N into milk protein [16], and reducing the amount of N excreted from the cow as MUN. However, reduced MUN per cow might physiologically be accomplished by reducing voluntary dry-matter intake, which would reduce metabolizable energy available above maintenance, and consequently reduce productivity per cow [20].

The national breeding objective in New Zealand is to generate dairy cows that are able to efficiently convert feed into profit. To achieve that objective, cows and bulls are selected based on the breeding worth (BW) index, which is calculated as the sum of the product of the estimated breeding values and respective economic value of each of the traits under selection. The BW index ranks the animals in units of net profit expressed in dollars per 5 tonnes of dry matter intake (DMI). The calculation of BW includes eight traits; lactation yields of milk, fat and protein, cow liveweight, fertility, longevity, body condition score, and somatic cell score [21]. Since MUN is associated both with N excretion [14] and N utilization efficiency [22], MUN may be considered as a candidate trait to include in the selection criteria for New Zealand dairy cattle to improve environmental sustainability and food security.

There are no published studies that have considered the response to selection from using MUN as a trait in the breeding objective. The objectives of this study were (1) to evaluate the correlated responses in lactation yields of milk (MY), fat (FY), crude protein (CPY), MUN and average mature cow liveweight (LWT) from three different selection indices in New Zealand dairy cows milked twice a day based on a conventional progeny test scheme, and (2) to evaluate the likely phenotypic correlated responses in production and UN per cow and per hectare for the three selection indices.

## 2. Materials and Methods

### 2.1. Base Cow

Average whole lactation MY, FY, CPY and average LWT of the New Zealand national dairy cows’ population were obtained from New Zealand Dairy Statistics 2018–2019 [23] to parameterize the base cow. The values obtained were annual yields of 4290 kg milk, 214 kg fat, 167 kg protein, and 456 kg LWT. Average MUN concentration during the lactation was assumed to be 14 mg/dL [19]. Milk urea yield (MUY; kg of milk urea produced) was calculated as milk urea (MU; mg/dL concentration of urea in milk) × MY where MU was calculated as MUN × 2.14 [24].

### 2.2. Lactation Curves

Although average whole lactation production of MY, FY, CPY, MUN and LWT of national dairy cows’ population was known, the average whole lactation excretions of UN and FN of those cows were unknown. The level of excretion depends on the level of intake and utilization of N by the cows. The yield of milk constituents and LWT gain/loss at each day of lactation by cows vary over time therefore, the energy requirement, DMI and involuntary N intake of the cows vary at each day of lactation. Consequently, lactation curves for MY, FY, CPY, MUN and LWT were simulated to estimate the daily DMI and involuntary N intake thereby lactation curves for UN and FN were simulated to estimate the annual UN and FN excretions.

Herd-test records of 634 cows with MY and percentages of fat (FP) and crude protein (CPP) were collected monthly from two research farms at Massey University and used to model the lactation curves of those traits. Fat yield and CPY were calculated from FP and CPP multiplied by the corresponding MY obtained during the herd-tests. MU concentration was determined indirectly for each cow for three of the herd-tests per season, representing early (September), mid (December) and late (March) lactations. Milk samples were assessed for MU at MilkTestNZ (Hamilton, NZ) using the CombiFossTM 7 instrument (Foss Electric, Hillerød, Denmark) based on a mid-infrared technique and not by colorimetry. A daily composite sample of morning and afternoon milking followed by weighting of milk according to morning and afternoon milk yields were used for estimating daily MUN concentration when twice a day milking was practiced, whereas the raw milk sample was used when once a day milking was practiced on the sampling days. Live weight measurements were generated at every herd-test using an automatic walkover weigh scale. Live weight change was calculated over the lactation as the difference in LWT at consecutive weightings. The Wilmink function [25] was used to model the lactation curves for MY, FY, CPY, LWT, and MUN as follows;

y_t_ = a + be^−kt^ + ct, (1)

where y_t_ indicates the predicted value at day t of the lactation, a, b, and c indicate the production (or live weight) after calving, the slope associated with production (or live weight) before the lactation peak, and slope associated with production (or live weight loss) after the lactation peak, respectively. The parameter k is associated with the time of lactation peak and was assumed to be −0.05 in this study, e is the base of the natural logarithm. The regression coefficients: a, b, and c estimated from the 634 cows using the Wilmink function were modified to obtain the total lactation yields of milk, fat, protein, and average across lactation MUN and LWT of cows under each scenario using Solver add-in in Excel [26] so that the sum of the squared differences between the predictions and actual productions were minimized. Daily metabolizable energy requirements for maintenance and lactation of the cow were estimated following the guidelines of the advisory manual prepared by the AFRC technical committee [27]. Apparent daily DMI was calculated as the estimated daily total metabolizable energy requirement of cow divided by total dietary metabolizable energy content of the pasture fed to cows that particular day. Daily crude protein intake of the cow was determined as dietary crude protein percentage available in any diet offered to the cow multiplied by the estimated daily DMI. Daily N utilization efficiency (NUE) was estimated as the proportion of N in CPY relative to estimated daily N intake (IN). Stocking rate (SR; defined as number of cows grazing per hectare), was estimated assuming an annual consumption of 12,000 kg of DM per hectare [28] divided by the estimated per cow annual requirement of DM. The annual per hectare productions were estimated as per cow annual productions multiplied by SR.

The daily UN excretion (g/d) for the lactation period (from day 1 to day 270) was estimated using the following equation developed by Huhtanen et al. [29]:

UN = −29 + (4.3 × DMI) + (4.3 × MUN) + (0.14 × LWT),(2)

and fecal N (FN, g/d) for the lactation period (from day 1 to day 270) was estimated as the N balance between intake and outputs (urine, milk, retention) using the following equation:

FN = IN − UN − MN − RN,(3)

where IN indicates grams of N intake, estimated as crude protein intake/6.25 (N percentage in protein), MN indicates grams of milk N, estimated as CPY/6.25, and RN indicates grams of retained N, estimated as liveweight change × 0.16 (each kg of liveweight change contains 0.16 kg of crude protein (CP) [29]).

The equations for estimating UN and FN rely on milk N, consequently, those equations are not directly suitable for use during the non-lactating winter period. The daily UN excretion from day 271 to day 365 was estimated by rearranging the above equation and with MN = 0 as described by Reed et al. [30]:

UN = IN − FN − RN.(4)

In order to estimate the UN excretion using the above equation, the FN from day 271 to day 365 must be known and that was estimated using the following equation [30]:

FN = 72.7 − (11.8 × ME) − (0.4 × NDF) + (3.5 × DCPP) + (0.2 × ForR) + (9.3 × DMI),(5)

where ME, NDF, and DCPP are megajoules (MJ) of metabolizable energy per kg of DM, percentage of neutral detergent fiber, percentage of crude protein content of diet, respectively. ForR is the percentage of forage in the ration, which was assumed to be forage only (100%) at each day of lactation.

The annual UN and FN excretions per cow were estimated as the sum of daily excretion (from day 1 to 365) of UN and FN, respectively, and the total N excreted was estimated as the sum of UN and FN excretions. The annual per hectare excretions were estimated as per cow annual excretions multiplied by SR. The total national excretion was estimated as the annual per hectare excretion times the total effective hectares of dairy land in New Zealand: 1.74 million ha [31], assuming that the effective land under dairy would not change in future. The across New Zealand annual change in N excretion was estimated as the difference between total across New Zealand excretion of the base cow (base year) and across New Zealand excretion under each scenario, divided by ten (the time difference between base year and each scenario presented was ten years). All UN and FN excretions were estimated assuming rate of excretion of national dairy cows’ population is the same as the cows were assumed to be solely fed on pasture diets.

Cows were assumed to be solely fed a pasture diet throughout the year, mainly containing ryegrass (*Lolium perenne*) and white clover (*Trifolium repens*). The dietary metabolizable energy [32], dietary crude protein percentage [32], and neutral detergent fiber [33] contents of the pasture were taken from published literature as representative of New Zealand pasture and used for estimating DMI and crude protein intake of cows. Published literature reported the monthly averages of dietary metabolizable energy, dietary crude protein percentage, and neutral detergent fiber, therefore, the quality of the pasture changed each month but was assumed to remain unchanged within each month (Table 1).

### 2.3. Breeding Scheme

The breeding scheme modelled in the study does not represent the current New Zealand breeding scheme which uses genomic selection to select bulls and bull dams for all the traits included in the selection index. Instead, this study models a traditional progeny testing methodology to provide an insight into the direction of responses for traits if MUN is included as a trait of interest in the selection index.

The contributions of each bull and cow selection pathway were separately evaluated to obtain the overall rate of genetic gain using four pathways of selection proposed by Rendel and Robertson [34], based on the differences in the generation intervals, and sources of information between males and females. This study assumed the New Zealand national herd comprised only one breed although in practice the New Zealand national herd comprised 4.95 million cows mainly made of two breeds and their crosses: Friesian (F), Jersey (J) and F and J cross-bred (F × J) [31]. From the national herd of 4.95 million cows, 90% were selected for producing cow replacements in cows to cows (CC) pathway. The total bulls available for progeny testing were assumed to be 300. From the total cow population, 1 million cows were assumed to be potential bull dams. The number of cows selected as active bull dams in cows to bulls (CB) pathway were 2100 assuming seven contract matings are required to produce one bull entering progeny testing (7 × 300). The best 10% bulls were selected for producing replacement cows, whereas 3% of elite bulls were selected for producing bull replacements in bulls to cows (BC) and bulls to bulls (BB) pathways, respectively. Cows were evaluated based on one of their own lactation records and bulls were evaluated assuming 75 half-sib progeny records. The generation intervals (L) were 4.8 (CC), 4.0 (CB), 6.6 (BC), and 6.3 (BB) years for each pathway. Generation interval for CC pathway, was estimated assuming replacement heifers from all age groups were retained for milking and majority of cows being selected in CB pathway are four years old. Generation intervals for bulls in BC and BB pathways were assumed considering the time required for obtaining progeny records from 75 daughters. Intensity of selection (i) and generation interval of each pathway are presented in Table 2.

### 2.4. Breeding Objective and Selection Index

The current New Zealand breeding objective comprises eight traits. This study evaluated the effect of inclusion of selection for or against MUN on a slightly simpler selection index comprised only of four key traits: MY, FY, PY, and LWT.

The economic values for MY, FY, PY, and LWT were as published by New Zealand Animal Evaluation Limited [21]. An economic value for MUN is zero as it is not included into the current breeding objective of New Zealand. Therefore, two economic values for MUN were assumed compared to zero and these were 20% relative emphasis with either a positive (MUN_+20%_) or a negative (MUN_−20%_) economic value on MUN. Relative emphasis on traits was calculated by multiplying the absolute economic value by the genetic standard deviation of the traits. Accordingly, three selection scenarios were performed: scenario 1 was to select with zero relative emphasis for MUN (MUN_0%_), genetic response for MUN was obtained from the genetic covariances with other traits, whereas scenario 2 and 3 were with 20% for MUN, positive (MUN_+20%_) or negative (MUN_−20%_) economic value for MUN, respectively. The economic values published by DairyNZ [28] and their relative emphasis on the traits under three scenarios are presented in Table 3.

### 2.5. Calculation of Selection Index and Prediction of Breeding Objective

The calculations of the selection index including estimation of ccorrelated responses and accuracy of the selection index were carried out as described by Cameron 1997 [36]. The selection index coefficients of multi-trait selection index [37] for each pathway were calculated using the following equation:

(6) $${b\;=\;P}^{-1}G\;a$$

where P is a 5 × 5 matrix of the phenotypic (co)variances between the traits in the selection index, and G is a 5 × 5 matrix of the genetic covariance between the traits in the breeding objective and those used for selection, and a is a 5 × 1 vector of economic values.

The matrices **P** and **G** were estimated for cows and bulls separately. The diagonal elements of the matrix **P** contain the phenotypic variances ($P_{ii}$) of the traits and the off-diagonal elements contain the phenotypic covariances ($P_{ij}$) between trait *i* and *j*. The elements $P_{ij}$ were estimated using knowledge of phenotypic variances and phenotypic correlations between traits *i* and *j* as presented in Table 4. The elements of matrix **P** for cows’ pathways were as follows:

(7) $$P_{ii}{\;=\;\sigma }_{pii}^{2}$$

and

(8) $$P_{ij}{\;=\;\sigma }_{pij}$$

where $\sigma_{pii}^{2}$ and $\sigma_{pij}$ indicate phenotypic variances and covariances, respectively.

The diagonal elements of the matrix G contain the genetic variances ($G_{ii}$) of the traits and the off-diagonal elements contain the genetic covariances ($G_{ij}$) between traits in the selection index and breeding objective. $G_{ij}$ was estimated using genetic correlation and heritability of traits *i* and *j*.

The environmental variances $\left(\sigma_{eii}^{2}\right)$ and covariances $\left(\sigma_{eij}\right)$ between trait *i* and *j* were estimated as:

(9) $$\sigma_{eii}^{2}{\;=\;\sigma }_{pii}^{2}\;-{\;\sigma }_{gii}^{2}$$

and

(10) $$\sigma_{eij}\;=\;\frac{r_{pij}\;-{\;(h}_{i}h_{j}r_{gij})}{{1\;-\;h}_{i}{\;h}_{j}}$$

where $\sigma_{pii}^{2}$, $\sigma_{gii}^{2}$, $r_{pij}$, $r_{gij}$ and h are phenotypic and genetic variances, phenotypic and genetic correlations between traits *i* and *j* and the square root of heritabilities, respectively, and the elements of the matrix **P** for bulls’ pathways evaluated using n number of daughters were constructed as:

(11) $$P_{ii}\;=\;\frac{((1\;+\;\left(n\;-\;1\right){\;t\;\sigma }_{gii}^{2})}{n}\;+\frac{\;\sigma_{\mathrm{eii}}^{2}}{n}$$

and

(12) $$P_{ij}\;=\;\frac{((1\;+\;\left(n\;-\;1\right){\;t\;\sigma }_{gij})}{n}\;+\;\frac{\sigma_{eij}}{n}$$

where t is the correlation among individuals of the progeny which is 0.25 for half-sib progeny. The elements of **G** matrix for bulls’ pathways were estimated as:

(13) $$G_{ii}{\;=\;k\;\sigma }_{gii}^{2}$$

and

(14) $$G_{ij\;}{=\;k\;\sigma }_{gij}$$

where k indicates relationship between sire and progeny which is 0.5 for bull to daughter [36].

Genetic parameters required for constructing **P** and **G** matrices were extracted from published literature [38,39] and are presented in Table 4. The **P** and **G** matrices were positive definite.

The overall response to selection is the sum of the selection responses in each individual trait. The correlated response (CR) of each trait to selection on BW was calculated as described by Cameron 1997 [36] using the following equation:

(15) $${\mathrm{CR}}_{j}\;=\;i_{I}\;\frac{b^{’}G_{j}}{\sqrt{b^{’}P}b}$$

where ${\mathrm{CR}}_{j}$ is CR of the *j*th trait and $G_{j}$ is the *j*th column of the **G** matrix and $i_{I}$ is the standardised selection differential in the selection index.

Total correlated response was calculated as the sum of correlated response in each pathway for each trait and then the total correlated response of each trait was divided by the sum of generation intervals of each pathway to estimate the annual rate of genetic gain in the breeding worth. The total economic response of the breeding objective (R_H_) is the sum of individual economic responses of each trait in the index. The R_H_ for each trait (MY, FY, CPY, LWT) was calculated as individual trait selection response multiplied by the respective relative economic value. However, MUN is not a component of the current New Zealand selection index for dairy cows and economic value of MUN is unknown therefore, MUN was excluded when estimating the correlated response in this study. The accuracy of the selection index (r_HI_) was calculated as:

(16) $$r_{\mathrm{HI}}\;=\frac{\sqrt{b^{’}P}b}{\sqrt{a^{’}G}a}$$

The correlated response, total correlated response, and $r_{\mathrm{HI}}$ for each trait under each scenario were estimated separately using four pathways of selection. The genetic gain of cows under each scenario for a period of ten years was expressed relative to the base cow by multiplying the estimated asymptotic annual genetic gain from each scenario by ten, assuming genetic gain is steady over generations, and this gain was added to the performance of the genetic base cow estimated in Section 2.2 to express the expected average performance of selected cows after ten years of selection.

## 3. Results

The predicted correlated response per cow per year in the assumed dairy cow population under three different scenarios based on the progeny test scheme are presented in Table 5. The predicted annual responses of the current selection index on a per cow basis were 16.4 kg MY, 2.0 kg FY, 1.4 kg CPY, −0.4 kg LWT, and −0.05 mg/dL MUN, respectively. The predicted increase of MUN for the positive selection index (MUN_+20%_) was 0.1 mg/dL with correlated increase of MY, FY, PY, and LWT. Applying a negative selection (MUN_−20%_) in the index resulted in a reduction of MUN, with increased MY, FY, PY, and reduced LWT. Selection of cows under positive economic values on MUN reduced the overall annual economic response of cows by 15% (NZD 1.80/cow), due to increased MY and LWT, whereas a negative economic values reduced the overall annual economic response by 11% (NZD 1.30/cow) due to reduced milk constituent yields when compared to the cow selected under the current index (MUN_0%_).

**Figure 1:**
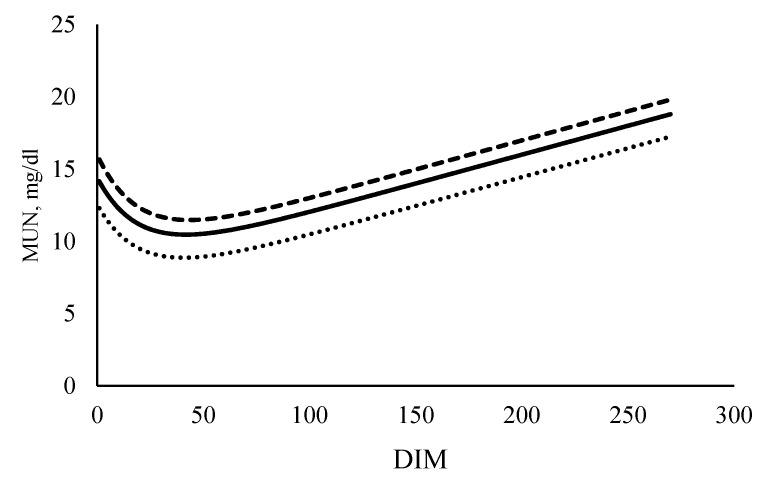
Concentration of milk urea nitrogen (MUN) at each day in milk (DIM) for cows in the base year (▬▬) and after ten years of selection with 20% relative emphasis and positive (MUN_+20%_) (**------**) or negative (MUN_−20%_) (‥‥‥) economic values for MUN.

The simulated performance for milk traits, liveweight and MUN after 10 years of selection under the three selection scenarios on a per cow and per hectare basis are presented in Table 6. The traits those were not included in the selection index under each scenario were estimated using the formulae presented in materials and methods section. The daily concentrations of MUN for cows in the base year and cows selected under positive and negative scenarios are shown in Figure 1.

After ten years of selection with the current selection index (MUN_0%_), there would be an increase of MY, FY, PY, a decrease of LWT along with a marginal decrease in MUN (0.55 mg/dL) per cow, compared to the base year. Ten years of selection under a positive scenario would increase MY, FY, PY, LWT and MUN per cow, compared to the base year. The selection based on the negative scenario for ten years would decrease MUN, compared to the base year, with per cow increase in MY, FY, PY, and decrease of LWT.

A loss of FY, CPY and LWT under negative selection (MUN_−20%_) resulted in a reduction in metabolizable energy requirements and therefore a subsequent reduction in DMI per cow compared to the MUN_0%_. The reduction in DMI per cow led to an increase in SR from 2.865 (MUN_0%_) to 2.914 cows/ha for equivalent utilization of pasture. Even though a similar production of fat and crude protein was observed under both MUN_0%_ and MUN_+20%_, the increased MY and LWT increased the metabolizable energy requirement which is reflected in the DMI of the cow in the latter scenario. This led to a need to reduce the SR under MUN_+20%_ relative to SR of the base year.

Ten years of selection based on a selection index with no emphasis on MUN will result in cows excreting 1.3 kg less UN and 6.7 kg more FN compared to the cows in the base year. The total N excreted (UN + FN) by the cows in base year is 112.8 kg which is predicted to be increased by 5.4 kg per cow as a consequence of MUN_0%_ selection scenario relative to cows in the base year. On a per ha basis the annual UN excretion is 168.6 kg and total N excreted is 336.5 kg in the base year and UN was estimated to reduce by 10.4 kg while N excreted was estimated to increase by 2.5 kg for cows selected with no emphasis on MUN.

The cows selected under the MUN_+20%_ for ten years would excrete 1.6 kg more UN, 5.7 kg more FN and 7.3 kg more total N compared to the cow in the base year. On a per hectare basis, due to the change in stocking rate, there will be a reduction of 3.5 kg of UN excretion with an increase of 4.6 kg of total N excretion compared to the base year. This means that by selection, the cows were manipulated to alter the N path.

After ten years of selection, a cow in MUN_−20%_ would have a reduced UN of 3.5 kg, with an increase of 7.9 kg in FN compared to cow in base year. This selection will also be responsible for 14.1 kg less UN but 5.1 kg more total N excretion on a per hectare basis compared to the base year. The total N excretion by cows selected under the MUN_+20%_ was slightly lower than that of the cow selected under MUN_−20%_.

Across New Zealand (over 1.74 m of effective hectares of dairy lands) the annual total N excretion (UN + FN) of cows in the base year was 585.5 m kg and was predicted to annually increase by 0.4 m kg, 0.8 m kg, and 0.9 m kg for cows selected under MUN_−0%_, MUN_+20%_, and MUN_−20%_, respectively.

**Figure 2:**
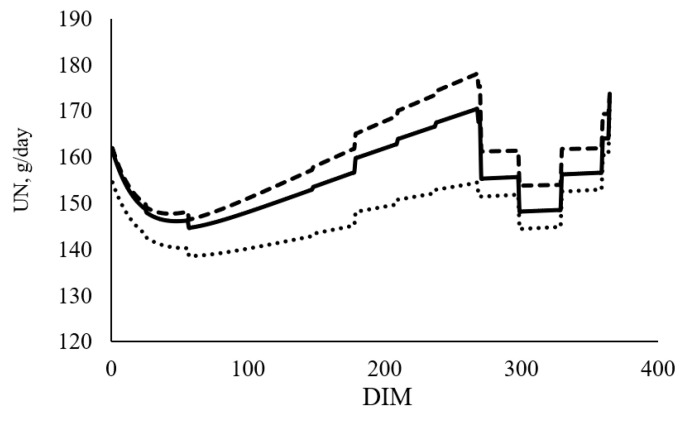
Nitrogen allocation for urinary nitrogen (UN) at each day in milk (DIM) in cows of base year (▬▬) and cows selected for ten years with 20% relative emphasis and positive (MUN_+20%_) (**------**) and negative (MUN_−20%_) (‥‥‥) economic values for milk urea nitrogen (MUN).

The across lactation average estimates of N allocation among the different N pools (MN, UN, FN) and NUE for cows in the base year and each selection scenario are presented in Table 6. A greater daily N allocation for MN and FN but lower daily N allocation for UN was predicted in the cow selected under the MUN_−20%_ (Figure 2), compared to the base year, whereas the N allocation for all three pools were higher in MUN_+20%_ scenario compared to the base year. There is only a slight NUE difference between cow selected under MUN_+20%_ and MUN_−20%_. The NUE of cows reduces with increased NI.

## 4. Discussion

The objective of the current study was to evaluate the effect of including MUN into the New Zealand BW index in order to reduce the UN excretion on a per cow and per hectare basis.

The predicted annual UN excretion per cow of the base year (56.5 kg) in the current study was near the range (74.8–91.6 kg) reported by Box et al. [40] in pasture-fed New Zealand dairy cows. The annual FN production of New Zealand dairy cows estimated using collected fecal samples in pasture-based feeding trials varies from 41.6 kg [41] to 48.5 kg [42] and are comparable with the estimated FN excretion in the base year (56.3 kg/cow/year) of the current study, which was estimated as the balance between the dietary IN and the other N outputs. The simulated annual MN production of the cow in base year in the current study was 26.7 kg and is within the range of MN production of pasture fed New Zealand dairy cows reported by Mackle et al. [43] (25.1–27.3 kg) and was comparable with values reported by Totty et al. [44] (27.3–28.1 kg). MPI, 2010 [45] reported the annual total N excretion per dairy cow in 2007 as 113.6 kg and the total annual N excreted by the New Zealand dairy cattle population (5.26 million) as 598 million kg. The annual total N excretion per cow and across New Zealand total excretion based on the SR and effective hectares for milk production in the 2018/19 production season (over 1.74 million ha) in the base year were 112.8 kg/cow and 586.5 million kg/ha, respectively. The estimated annual NI (142 kg) of the base cow as determined from the predicted DMI of this study is similar to the NI (166 kg) estimated using measured DMI in 24 J and F cows by Mackle et al. [43]. The estimations of N partitioning among different pools, DMI, NI and excretions of the predicted base cow of the current study appear representative of a New Zealand cow. Therefore, the predicted base cow in this study is an appropriate reference point to compare different selection scenarios.

In this study the genetic response to selection is taken at the asymptotic point. However, after the mating of selected dams and bulls, the new-born calves which are genetically capable of producing less MUN will be included into the age group zero after calving and will not produce milk with less MUN until they calve for the first time at two years of age. This means there will be at least three years of lag with no improvement in milking cow phenotype therefore immediate results cannot be expected through selection.

The correlated responses in FY and CPY predicted for the MUN_0%_ selection scenario in the current study were comparable with those reported in Sneddon et al. [45] and Spelman and Garrick, [38], and the simulated correlated responses for MY and LWT for the MUN_0%_ were comparable with those reported in Spelman and Garrick, [38].

Negative selection for MUN was responsible for the greatest reduction in MUN concentration, however, that scenario had unfavorable correlated responses for FY and CPY due to positive correlation of MUN with FY and CPY. That resulted in less improvement of profit compared to the current index, because payment for milk in the New Zealand dairy industry is based on the A + B − C multiple component pricing system, where A and B are the values per kg of fat and protein and C is the penalty on kg of MY [46]. Selection for increased MUN (MUN_+20%_) was responsible for the same milk solid production as the MUN_0%_. Since yield of milk solids is highly sensitive to the negative economic value on MUN, selection against MUN should be considered with caution.

The number of milking cows in the 2018–2019 production season was 4.95 million [31] and if the national herd size remains unchanged, NZD 8.91 million (NZD 1.8 × 4.95) or NZD 6.4 million (NZD 1.3 × 4.95) income loss is expected annually after ten years of selection for the MUN_+20%_ and MUN_−20%_, respectively, compared to the cows selected under MUN_0%_. The reduced economic response in MUN_−20%_ will be caused by reduced genetic gains of FY and CPY which have positive economic values. The increased genetic gains of MY and LWT which have negative economic values are the likely reason for the reduced economic response in MUN_+20%_.

Although fertility traits have not been included in the selection index in this study due to unavailability of variance-covariance information, König et al. [42] reported negative genetic and phenotypic correlations between MUN and 56-day nonreturn rate (phenotypic = −0.08, genetic = −0.13) and 90-day nonreturn rate (phenotypic = −0.10, genetic = −0.12) in German Holstein cows, indicating that cow fertility would be improved if MUN was reduced. However, König et al. [47] reported that the genetic correlations between MUN and nonreturn rates were too weak to justify the use of MUN as an indicator trait in genetic selection for improved fertility. Therefore, correlations between MUN and reproduction traits need to be estimated for New Zealand dairy cows to define the selection index with production, reproduction, and environmental sustainability traits.

The annual per hectare UN excretion of cows estimated from per cow UN excretion and current SR (2.982) were 168.6 kg in the base year, 164.60 kg in MUN_0%_ (55.2 kg × 2.982), 173.3 kg in MUN_+20%_ (58.1 kg × 2.982), and 158.2 kg in MUN_−20%_ (53.0 kg × 2.982). This would result in an annual per hectare change of UN excretion of -4.0 kg in MUN_0%_, 4.7 kg in MUN_+20%_, and −10.4 kg in MUN_−20%_ compared to the base year. However, the cows selected under all the three scenarios had greater production of milk yield and milk solids compared to cows in the base year due to positive correlated responses observed for milk yield and milk solids. This required the selected cows to have a higher DMI and consequently there would be a lower SR than for cows in the base year. The MUN_0%_ and MUN_−20%_ allowed the cows to excrete less UN on a per hectare basis (−10.4 kg and −14.1 kg, respectively) than cows in the base year.

Most of the prediction equations for MUN and UN in the literature were derived under indoor conditions where cows were fed with TMR [48,49,50,51]. However, under New Zealand outdoor grazing conditions, DMI is controlled at the herd level, allowing individual cows to vary in voluntary feed intake, given the same access to pasture. Accordingly, the IN of cows in the same herd will exhibit variation that contributes to between cow variation in MUN and UN. Milk urea nitrogen is affected by various factors besides genetic makeup, including; level of production, level of protein feeding, and stage of the lactation and therefore, there are subsequent effects of those factors on UN. Given that MUN is a multifactorial trait, the prediction equations containing additional dependent variable other than MUN were used for estimation of UN in the current study, which is combination of equations predicted by Huhtanen et al. [29] and Reed et al. [30]. Huhtanen et al. [29] developed a prediction equation for UN in lactating cows that were fed indoors with forage, concentrates and TMR. In their equation, UN was estimated as a function of DMI, LW, and MUN where, DMI is a representative of both level of production and level of crude protein intake, LWT is a measure of level of production, and MUN is a measure of N partitioning on excretion. Therefore, the estimates of UN in their study was an indirect measure of balance between the IN and utilization and output. In indoor feeding trials, Reed et al. [30] derived a prediction equation for UN independent to MUN, directly as the balance between N intake, and N utilization and outputs (fecal N, N in milk, retention of N in the body: estimated as a function of liveweight gain or loss). The partitioning of dietary nutrients for different processes is genetically driven [52] and specific to each animal, therefore, it seems more appropriate to use these equations for estimating UN, as performed in our study, rather than a direct conversion of MUN into UN, as assumed by Beatson et al. [19].

Cows selected for higher MUN (MUN_+20%_) produced higher MUN on a per cow basis and excreted less UN (−3.5 kg) on a per hectare basis due to the reduced SR in comparison to the base scenario. This means that a base year cow with less MUN on a per cow basis (14 mg/dL), excreted more UN (168.6 kg) on a per hectare basis compared to a cow with higher MUN (14.9 mg/dL) on a per cow basis but less UN per ha (165.1 kg). The simulated result of the current study indicates that the increased MUN at per cow basis is not necessarily responsible for reduced UN on a per hectare basis because a lower SR plays an important role in reducing UN on a per hectare basis. A recent New Zealand study [53] has indicated that there is less opportunity to reduce N leaching by genetic selection for urine traits (total volume of urine per cow per day and average volume per urination) when SR and IN of cows are high. The results from this simulation study, however, contradict the points made by Roche et al. [20] who indicated that N leaching can be reduced with increased SR with minimum supplementary feeding. The reduced N excretion with increased SR in their study is reasonable due to per cow reduction of DMI hence, reduced involuntary IN of cows whereas, in the current study DMI is increased with increased milk production of cows by reducing the SR which allowed UN to reduce.

Selected cows in all three scenarios excreted higher FN compared to the cows in the base year. These cows also had greater N allocation for MN due to the increased production of CPY compared to the cows of the base year. These simulated results concur with Marshall et al. [54] who reported that cows with low MUN BV had increased milk protein percentage throughout lactation and FN in late lactation. The higher N allocation for other N pools, apart from urine, is likely be linked with higher involuntary IN associated with higher DMI of genetically improved cows. However, the increased FN allocation of improved cows made cows selected in all three scenarios equivalent in terms of the total N excreted, despite the relative emphasis and direction of selection for MUN.

The simulated daily reduction of UN as reported by Beatson et al. [19] was 18 g/cow per year of selection in the progeny of the low MUN breeding value (BV = −2.4) bulls compared to the progeny of bulls with MUN BV = 0. Low MUN cows had a mean phenotype of 14.0 mg MUN/dl (New Zealand average) for MUN. This reduction in UN corresponds to an annual reduction of 6.6 kg/cow and that estimate is supported by the study of Marshall et al. [54] using measured urinary urea N excretions in 58 multiparous, lactating Holstein-Friesian cows. Beatson et al. [19] calculated that this reduction in UN excretion corresponded to an annual reduction of 42 million kg N from the 6.5 million dairy animals farmed across New Zealand from already published prediction equations for estimating UN based on MUN. According to the estimates in this study, the correlated response of MUN after ten years of selection in the negative scenario (MUN_−20%_), and using a combination of prediction equations of Huhtanen et al. [29] and Reed et al. [30], there would only be a reduction of 17.3 million kg of UN over the 4.95 million of milking cows (3.5 kg/cow/ten years × 4.95 million cows). Following the same assumptions, MUN was predicted in the current study after ten years of selection under MUN_+20%_, reporting 7.9 m kg (1.6 kg/cow/ten years × 4.95 million cows) of increase in UN excretion. Difference in UN excretion estimates between Beatson et al. [19] and this study is likely due to differnce in prediction equations used.

There is a positive relationship between IN and UN outputs in New Zealand dairy cows [42,42]. New Zealand pasture on average contains around 200 g of CP/kg DM (20%) [55] but, is even richer in CP (265 g/kg DM = 26.5%) during the spring [56]. The current study evaluated the possible improvement of UN excretion using a progeny test-based selection scheme under typical New Zealand feeding management, where cows fed on diets containing CP ranging from 190 to 240 g/kg DM, and assuming a positive relationship between MUN and UN. On the contrary, a recent study conducted based on German Holsteins revealed that the relationship between MUN and UN was positive only if the cows were fed with low CP diets (13.8%) while it was negative for normal CP (15.9%) diets [57]. This finding suggests that for New Zealand cows which consume such great CP %, they are less likely to reduce UN through genetic selection for reduced MUN, which means that diet management is the likely best alternative to control UN excretion and carbon footprint.

Some authors have reported a negative relationship between MUN and NUE [57] and a negative relationship between NUE and IN [18,58]. Therefore, it is plausible that by including MUN into the selection index with a negative economic value that the correlated response of MUN would result in cows with improved NUE. Although NUE increased in the MUN_−20%_, at reduced NI in the current study, the level of production including volume and milk constituents were also reduced compared to cows selected under the MUN_0%_. The reduction of milk and milk constituent yields are explained by the strong positive genetic correlations between yields of milk constituents and milk volume [39,59] and positive genetic correlation between MUN and volume of milk [19,38]. This attenuation of milk constituent production is not desirable in terms of farm profitability, because price of milk in New Zealand is a function of milk constituents.

Considering the overall economic impact to the New Zealand dairy industry, neither the negative (MUN_−20%_) nor the positive (MUN_+20%_) selection scenarios were favorable compared to the MUN_0%_, owing to the reduced production of milk constituents and increased MY and LWT, respectively. However, the economic value of MUN was not considered when estimating the overall economic responses under the selection scenarios due to uncertainty of the true economic value for MUN.

Aggregate breeding values in the current selection scheme for the genetic improvement of New Zealand dairy cattle combines genomic, performance and pedigree information of animals for all traits. Schefers and Weigel, [60] documented the advantages of including genomic selection as a selection strategy and demonstrated that the genetic change per year with genomic selection is double that of the genetic change per year achieved through a conventional progeny test selection scheme. However, setting up the P and G matrices for a combined breeding goal comprised of multiple traits, considering the covariance between performance records and genotypes for all the traits is complex [61] and relies on many assumptions, and so this approach was not investigated in the current study. Instead, standard selection index theory [62] based on a conventional progeny test was simulated to determine responses to various selection indices including MUN. Although the correlated response might have been improved if genotypic information were incorporated due to the reduced generation interval, the direction of response to selection is unlikely to change as it depends on genetic variation of the trait and the genetic correlation with other traits in the index [63].

## 5. Conclusions

Selection for MUN through the conventional breeding programs had no substantial benefit in reducing UN excretion in grazing dairy cows of New Zealand. The correlated response when applying a negative economic value for MUN is not favorable for fat and protein yields, and the income of the farmers would be negatively affected due to reduced milk constituents in attempting to reduce UN. Compared to the current selection index, the selection of bulls for low MUN breeding values would cause the New Zealand national dairy herd to comprise cows with lower Breeding Worth, thereby affecting future milk fat and protein production of the country. Cows with the genetic potential to produce greater MUN do not necessarily increase N available for leaching prior to pasture uptake on a per hectare basis. This is because N leaching is positively associated with SR and SR needs to be reduced with increased DMI of genetically improved cows to ensure the cows receive adequate ME. Consequently, the UN excretion of cows selected with positive economic value for MUN is reduced on a per hectare basis. However, the predicted reduction in UN excretion per cow is marginal even under greater negative relative emphasis for MUN, in addition results are not realized until several years into the future. Based on the assumptions applied in this study, total N excreted is unable to be controlled by placing either negative or positive selection pressure on MUN. Therefore, other options such as feeding cows with diets balanced for crude protein relative to energy and protein feeding based on their requirements should get more attention for controlling N excretion. Additional studies are needed to make a comprehensive evaluation of the effect of including MUN in the current New Zealand BW index.

## Figures and Tables

**Table 1:** Assumed quality of the pasture including dietary metabolizable energy (DME), dietary crude protein percentage (DCPP), and neutral detergent fibre (NDF) used for estimating dry matter intake, protein utilization efficiency and faecal nitrogen excretion in the current study.

	Pasture Quality		
Month	DME ^1^ (MJ ME/kg DM)	DCPP ^1^ (%)	NDF ^2^ (%)
Jun	11.0	24	35
Jul	11.0	24	35
Aug	11.1	22	34
Sep	11.4	24	35
Oct	11.4	23	37
Nov	11.4	21	41
Dec	11.3	19	42
Jan	10.7	21	44
Feb	10.5	22	44
Mar	10.4	21	41
Apr	11.0	23	38
May	11.0	22	37

^1^ Litherland and Lambert, [32]; ^2^ Moller et al., [33].

**Table 2:** Assumptions made for performing four pathways of selection for improving genetic gain of New Zealand dairy cows.

Path	Population	Number Selected	Proportion Selected	i	L	Number of Records	Numberof Progeny
CC	4,900,000	4,410,000	0.90	0.20	4.8	1	0
CB	1,000,000	2100	0.002	3.20	4.0	1	0
BC	300	30	0.10	1.76	6.6	0	75
BB	300	9	0.03	2.27	6.3	0	75

CC, cows to cows; CB, cows to bulls; BC, bulls to cows; BB, bulls to bulls; i, intensity of selection; L, generation interval (years).

**Table 3:** Economic values (EV) and relative emphasis for traits in three different selection scenarios that included milk urea nitrogen (MUN) with different relative emphasis in the breeding objective; zero (MUN _0%_), twenty percent with positive (MUN_+20%_) or negative (MUN_−20%_) EV.

	Breeding Objective					
	MUN _0%_		MUN_+20%_		MUN_−20%_	
Trait ^1^	EV	RE	EV	RE	EV2	RE
MY, kg	−0.09	20.22	−0.09	16.18	−0.09	16.18
FY, kg	3.49	31.66	3.49	25.33	3.49	25.33
CPY, kg	4.38	29.24	4.38	23.39	4.38	23.39
LWT, kg	−1.30	18.87	−1.30	15.10	−1.30	15.10
MUN, mg/dL	0.00 ^‡^	0.00	24.35 ^‡^	20.00	−24.35 ^‡^	20.00

^1^ MY, milk yield; FY, fat yield; CPY, crude protein yield; LWT, liveweight; MUN, milk urea nitrogen. EV, economic value obtained from the DairyNZ [21]; RE, relative emphasis calculated by multiplying the absolute economic value of each trait by the corresponding genetic standard deviations and divided by the sum of the absolute values of these products, then multiplied by 100 [35]; ^‡^ Economic value calculated based on the RE assigned to MUN in the breeding objective.

**Table 4:** Estimates of genetic parameters^1^ for yields of milk (MY), fat (FY), crude protein (CPY), and for liveweight (LWT) and milk urea nitrogen (MUN) as used in selection index.

				Correlations ^1^				
Trait	h^2^	t	σ_p_	MY	FY	PY	LWT	MUN
MY	0.28 ^a^	0.60 ^a^	519.52 ^a^		0.80 ^a^	0.90 ^a^	0.20 ^a^	0.11 ^b^
FY	0.22 ^a^	0.60 ^a^	22.61 ^a^	0.60 ^a^		0.90 ^a^	0.18 ^a^	0.02 ^b^
CPY	0.25 ^a^	0.60 ^a^	16.64 ^a^	0.80 ^a^	0.70 ^a^		0.22 ^a^	0.05 ^b^
LWT	0.24 ^a^	0.65 ^a^	34.64 ^a^	0.39 ^a^	0.34 ^a^	0.37 ^a^		0.02 ^c^
MUN	0.22 ^b^	0.35 ^b^	2.340 ^b^	0.19 ^a^	0.04 ^b^	0.08 ^b^	0.31 ^c^	

h^2^, heritability; t, repeatability; σ_p_, phenotypic standard deviation. ^1^ Genetic correlations below diagonal and phenotypic correlations above diagonal. ^a^ Spelman and Garrick [38], ^b^ Beatson et al. [19], ^c^ Lopez-Villalobos et al. [39].

**Table 5:** Annual correlated responses in five traits using different selection scenarios that include milk urea nitrogen (MUN) with different relative emphasis in the breeding objective, zero (MUN_0%)_ and twenty percent (with positive; MUN_+20%_ and negative MUN_−20%_ economic values).

	Breeding Objective		
Trait ^1^	MUN_0%_	MUN_+20%_	MUN_−20%_
MY, kg	16.4	23.7	5.4
FY, kg	2.0	2.0	1.6
CPY, kg	1.4	1.4	1.0
LWT, kg	−0.4	0.6	−1.1
MUN, mg/dL	−0.05	0.1	−0.17
R_H_	NZD 12.30	NZD 10.50	NZD 11.00

^1^ MY, milk yield; FY, fat yield; CPY, crude protein yield; LWT, live weight; MUN, milk urea nitrogen; R_H_, overall economic response; sum of individual trait genetic gain multiplied by their corresponding relative economic values only for milk, fat, protein and live weight.

**Table 6:** Expected average lactation yields of milk (MY), fat (FY), crude protein (CPY), liveweight (LWT) and milk urea nitrogen (MUN) in cows of base year and different selection scenarios after 10 years of selection for MUN with different relative emphasis in the breeding objective, zero (MUN_0%_), and twenty percent with positive (MUN_+20%_), and negative (MUN_−20%_) economic values.

		Breeding Objective		
Trait	Base Year	MUN_0%_	MUN_+20%_	MUN_−20%_
Per cow ^1^				
MY, kg	4290	4454	4527	4344
FY, kg	214	234	234	230
CPY, kg	167	181	181	177
LWT, kg	456	452	462	445
DMI, kg	4024	4188	4226	4119
MUN, mg/dL ^1^	14.00	13.45	14.90	12.33
MUY, kg	1.2	1.2	1.4	1.1
IN during lactation *, kg	125	131	132	128
IN, kg	142	148	149	145
MN, kg	26.7	29.0	29.0	28.7
FN, kg	56.3	63.1	62.0	64.2
UN, kg	56.5	55.2	58.1	53.0
Total N excreted, kg	112.8	118.3	120.1	117.2
NUE, %	21.4	22.2	22.1	22.3
Per ha ^2^				
SR, cows	2.982	2.865	2.840	2.914
MY, kg	12,792	12,762	12,855	12,656
FY, kg	638	670	664	670
CPY, kg	498	519	514	516
MUY, kg	3.5	3.5	4.0	3.2
IN, kg	423	423	423	423
MN, kg	79.6	83.2	82.3	83.5
FN, kg	167.9	180.8	176.1	187.0
UN, kg	168.6	158.2	165.1	154.5
Total N excreted, kg	336.5	338.9	341.0	341.6
Across New Zealand ^3^				
Total N excreted, million kg	585.5	589.8	593.4	594.3
Change in N excretion, million kg/year		+0.4	+0.8	+0.9

DMI, day matter intake; MUY, kilograms of milk urea yield; IN, nitrogen intake, MN, milk nitrogen; FN, fecal nitrogen; UN, urinary nitrogen; Total N excreted, sum of FN and UN; NUE, nitrogen utilization efficiency; change in N excretion, across country annual total N excreted in base year—across country annual total N excreted in each selection scenario. ^1^ Estimated using correlated response, ^2^ estimated as per cow estimate × SR, ^3^ estimated as per ha estimate × 1.74 million hectares (number of effective hectares of dairy lands). * Sum of IN only during the lactation period (from 1–270 days in milk); base year, year in which the selection index initiated.

## Data Availability

All relevant data are within the paper.
